# Navigating the Border - How Swedish Physicians Approach Palliative Care Referrals in Oncology

**DOI:** 10.1177/10732748251382299

**Published:** 2025-10-03

**Authors:** Karin Adolfsson, Ulrika Kreicbergs, Jan Mårtensson, Charlotte Bratthäll, Erik Holmberg, Thomas Björk-Eriksson, Margaretha Stenmarker

**Affiliations:** 1 Department of Oncology, Institute of Clinical Sciences, Sahlgrenska Academy, 70712 University of Gothenburg, Gothenburg, Sweden; 2 Department of Oncology, Jönköping, Sweden; 3 Great Ormond Street Institute of Child Health, 11700 University College London, London, UK; 4 Department of Nursing Science, School of Health and Welfare, 145651 Jönköping University, Jönköping, Sweden; 5 Department of Oncology, Kalmar, Sweden; 6 Department of Paediatrics, Institute of Clinical Sciences, Sahlgrenska Academy, 70712 University of Gothenburg, Gothenburg, Sweden; 7 Department of Paediatrics, Linköping University, Jönköping, Sweden; 8 Department of Biomedical and Clinical Sciences, 4566 Linköping University, Linköping, Sweden

**Keywords:** palliative care, oncology, cancer, referral and consultation, physicians, patients, organisations

## Abstract

**Introduction:**

Early integration of palliative care (PC) alongside oncology care is widely recognised as beneficial for patients and their next of kin. Sharing the responsibility with colleagues can ease the burden for the physician. However, according to our previous research with physicians, while two out of three expressed a positive attitude towards early integration of PC, only one-third actually implemented it. To facilitate the early integration of PC, the physicians’ own attitudes need to be highlighted. The aim of this study was to explore Swedish physicians’ personal experiences and their view of the role of the organisation when referring patients with cancer to PC.

**Methods:**

A study was performed using a study-specific questionnaire. Physicians working with cancer patients within different specialties participated. Data were collected in a cross-sectional online survey. Quantitative data (items) were analysed using descriptive statistics and open-ended responses were analysed with thematic analysis.

**Results:**

In total, 130 physicians participated. The majority reported feeling confident when introducing PC to patients (97.6%) They expressed a high degree of medical and emotional relief when the patients were enrolled in PC. Organisational challenges were reported in gaining acceptance from PC providers (50.9%) and in ensuring equal access to PC (54.5%). Thematic analysis identified multifaceted aspects related to navigating barriers and facilitators in the referral process, the benefits achieved from mutual collaboration with PC providers, and the physician related challenges when managing the organisational labyrinth.

**Conclusion:**

The physicians expressed confidence in discussing PC with patients. They experienced personal, professional, patient-related and organisational benefits when patients were enrolled in PC. A majority expressed that the patients did not have equal access to PC. To reach this goal, organisational aspects, including communication pathways and geographical restrictions, need to be addressed.

## Introduction

In the last few decades, there has been a rapid development of new therapeutic strategies for cancer, leading to an increasing number of patients living with advanced disease. These new treatment options have resulted in cancer now being considered a chronic disease, making prognostication of the disease course more challenging for treating physicians.^1,2^ According to the International Agency for Research on Cancer approximately 20 million individuals were diagnosed with cancer 2022 and an estimated 53.5 million people were living within 5 years of a cancer diagnosis. By 2050, the number of new cancer cases is projected to exceed 35 million globally.^3^ Furthermore, population aging presents a significant worldwide challenge, contributing to a growing demand for health care services, including oncological and palliative care (PC).^1^ PC, as defined by the World Health Organisation (WHO), is considered ‘applicable early in the course of illness, in conjunction with other therapies that are intended to prolong lifeʼ.^4^ A substantial amount of evidence supports the integration of PC alongside oncological care during the early stages for patients with advanced malignancies.^5,6^ This approach has also been recommended in international guidelines.^7,8^ However, the timing of referral to PC still predominantly relies on the judgement of the responsible oncologist.^9,10^ In clinical practice, patients are often referred late in the disease trajectory, when symptom control becomes challenging, when ending anti-cancer treatment or when transitioning to end-of-life care is imminent.^11^ Our previous study among Swedish physicians confirmed this practice.^12^ From an organisational perspective, PC can be provided at two levels of care. Primary PC can be described as a care requiring basic skills and knowledge, ie, expertise that every physician should have. Specialised PC deals with the treatment of more complex symptoms and needs.^13^ Earlier research has identified several barriers to PC referral. Organisational challenges include limited time available to referring physicians, differing opinions among healthcare providers, and insufficient resources or limited accessibility within PC services. Additional barriers involve prognostic uncertainty, the stigma associated to PC, and reluctance among patients and families.^10,11,14-16^ Facilitating factors can include renaming the service, communication skills and knowledge about PC among healthcare providers and in society.^9,14^

Focusing on the physician’s personal experiences of managing patients with advanced cancer can entail an emotional burden,^17^ and may be a demanding task when handling both anti-cancer therapy and referral to PC.^9,18^ However, the management can also be a source of satisfaction.^17^ Support from colleagues can be valuable in addressing challenges experienced in medical practice.^19,20^ Sharing the responsibility with PC physicians can alleviate the personal burden and thereby relieve the emotional and medical strain for the referring physician.^21^

Today, there are few European studies that highlight the topic of the process of PC referral, including barriers and facilitators in the referral process between oncological care and PC.^22^ The aim of this study was to explore Swedish physicians’ personal experiences and their view of the role of the organisation when referring patients with cancer to PC.

## Methods

### Study Design

A cross-sectional online survey with a study-specific questionnaire focusing on the referral process from oncological care to PC was performed. Quantitative data were collected using items with predetermined response options. In addition, the participants were asked to elaborate their answers through open-ended responses to items in the same study specific-questionnaire. The study design aimed to elicit rich, descriptive data and gain deeper understanding of physician’s personal experiences, as well as the perceived role of the organisation in shaping those experiences.

### Participants and Context

The participants included physicians working with patients suffering from malignancies within the medical specialities of gynaecological oncology/gynaecology, haematology, oncology, paediatric oncology, pulmonology and urology. These physicians were responsible for handling medical oncological treatment or radiotherapy. The exclusion criterion was physicians who were solely in charge of surgical oncological treatment. All participants worked within the south-eastern health region in Sweden. This region consists of three counties, with each county having its own healthcare organisation and management.

### Study-Specific Questionnaire

A study-specific questionnaire was designed based on a review of relevant literature,^5,6,23-25^ clinical experience and semi-structured interviews conducted with three senior oncologists. The interviews identified 5 main themes, which led to 5 dimensions presented below. A pilot study was conducted with six experienced physicians who worked with cancer patients to evaluate the questionnaire’s readability, clarity and content validity. Following feedback and subsequent revisions, the questionnaire was re-evaluated by three physicians. The final questionnaire consisted of questions covering demographic data and the following 5 dimensions: ‘Attitudes’ (n = 3), ‘Practices’ (n = 19), ‘Work-related experiences’ (n = 6), ‘Personal experiences’ (n = 12), and ‘The role of the organisation’ (n = 11). Options were presented on six-point Likert scales (ranging from 1 = ‘never’ to 6 = ‘always’ or 1 = ‘very negative’ to 6 = ‘very positive’), as well as multiple choice questions with options such as ‘yes’, ‘no’ and ‘I partly agree’ or ‘very good’, ‘good’, ‘acceptable’, ‘bad’ and ‘I do not know’. Qualitative data were collected through open-ended responses related to each one of the items. A more detailed description of the development of the questionnaire and the dimensions of ‘Attitudes’, ‘Practices’, and ‘Work-related experiences’ have been described in our previous publication.^12^ In the present study, we present the study characteristics (Table 1) and the dimensions of ‘Personal experiences’, as well as ‘The role of the organisation’ (Tables 2 and 3). The answer options “sometimes” and “often” are presented together, which also applies to the alternatives “yes” and “partly” and “very good” and “good”. The dimension of ‘Personal experiences’ covered aspects of personal reflections, patient-physician relations, knowledge and opinions about PC services. ‘The role of the organisation’ involved collaboration with PC services and colleagues and its impact on oncology care.

**Table 1.** Description of the Study Population (N = 130)

Characteristics	n (%)
Gender	
Female	71 (54.6)
Male	59 (45.4)
Medical specialty	
Gynaecological oncologist/gynaecologist	38 (29.2)
Haematologist	10 (7.7)
Oncologist	36 (27.7)
Paediatric oncologist	14 (10.8)
Pulmonologist	10 (7.7)
Urologist	22 (16.9)
Medical career	
Specialist/senior consultant	100 (78.1)
Resident	28 (21.9)
Missing	2
Experiences in treating cancer patients	
≤10 years	48 (40.7)
>10 years	70 (59.3)
Missing	12

N = 130 (population size), n = subset of N answering.

**Table 2.** The Physicians’ Personal Experiences (N = 130)

Personal reflections and patient/physician relations	Often n (%)	Sometimes n (%)	Seldom n (%)
When you introduce the concept of PC to a patient with cancer
a. Do you feel confident talking with the patient? (n = 125)	99 (79.2)	23 (18.4)	3 (2.4)
b. Are you afraid of making the patient upset? (n = 126)	14 (11.1)	40 (31.7)	72 (57.1)
c. Do you feel unease in the situation? (n = 126)	14 (11.1)	55 (43.7)	57 (45.2)
d. Do you feel that the patient still has trust in you? (n = 126)	106 (84.1)	19 (15.1)	1 (0.8)

	Yes n (%)	Partly n (%)	No n (%)
Do you experience medical relief if the patient is enrolled in primary PC? (n = 109)	42 (38.5)	54 (49.5)	13 (11.9)
Do you experience medical relief if the patient is enrolled in specialised PC? (n = 110)	71 (64.5)	31 (28.2)	8 (7.3)
Do you experience emotional relief if the patient is enrolled in primary PC? (n = 106)	29 (27.4)	50 (47.2)	27 (25.5)
Do you experience emotional relief if the patient is enrolled in specialised PC? (n = 108)	46 (42.6)	46 (42.6)	16 (14.8)

Knowledge about PC services	Yes n (%)	Partly n (%)	No n (%)
Do you know how PC services are organised in your county? (n = 126)	70 (55.6)	50 (39.7)	6 (4.8)
Do you ask your colleagues about the organisation of PC? (n = 123)	35 (28.5)	60 (48.8)	28 (22.8)
Do you use digital sources for information about PC organisation? (n = 125)	11 (8.8)	36 (28.8)	78 (62.4)
Do you know which criteria decide the level of care, primary or specialised PC? (n = 123)	43 (35.0)	58 (47.2)	22 (17.9)

Abbreviation: Palliative care (PC).

N = 130 (population size), n = subset of N answering.

Not all participants answered every question, valid percentages are presented.

**Table 3.** The Role of the Organisation (N = 130)

Early PC- influence on oncology care	Yes n (%)	Partly n (%)	No n (%)
Do you get information from the recipient that affects your choice of oncological treatment? (n = 108)	10 (9.3)	48 (44.4)	50 (46.3)
Is the choice of oncological treatment affected if the patient is enrolled in primary PC? (n = 104)	3 (2.9)	8 (7.7)	93 (89.4)
Is the choice of oncological treatment affected if the patient is enrolled in specialised PC? (n = 110)	3 (2.7)	15 (13.6)	92 (83.6)

Early PC – organisational challenges	Yes n (%)	Partly n (%)	No n (%)
Do you have difficulties in motivating the recipient about the patient’s need for enrolment in PC? (n = 108)	17 (15.7)	38 (35.2)	53 (49.1)
Do you actively obtain information from the recipient about the patient? (n = 108)	30 (27.8)	47 (43.5)	31 (28.7)

	Often n (%)	Sometimes n (%)	Seldom n (%)
Is there a clear distribution of responsibilities between you and the recipient? (n = 111)	34 (30.6)	63 (56.8)	14 (12.6)
Do you have an active dialogue with the recipient about the status of the patient? (n = 109)	22 (20.2)	54 (49.5)	33 (30.3)

Opinions about PC services	Yes n (%)	Partly n (%)	No n (%)
Is your opinion that all patients receive equitable PC independently of where they live? (n = 121)	19 (15.7)	36 (29.8)	66 (54.5)

	Very good n (%)	Good n (%)	Acceptable n (%)	Bad n (%)	Don’t know n (%)
How do you think that PC services are functioning overall in your county? (n = 126)	20 (15.9)	46 (36.5)	38 (30.2)	16 (12.7)	6 (4.8)
How do you think that primary PC services are functioning in your county? (n = 125)	9 (7.2)	36 (28.8)	41 (32.8)	21 (16.8)	18 (14.4)
How do you think that specialised PC services are functioning in your county? (n = 124)	40 (32.3)	52 (41.9)	14 (11.3)	12 (9.7)	6 (4.8)

Abbreviation: Palliative care (PC).

N = 130 (population size), n = subset of N answering.

Not all participants answered every question, valid percentages are presented.

### Data Collection

An online survey was performed using ‘esMaker’ (Version N3X, Entergate AB), a web-based system where data is automatically anonymised. Nine hospitals with 27 departments participated. The number of potential, eligible physicians was obtained by contacting the operational managers at these departments. A detailed description of the data collection process has previously been published.^12^ To identify the intended study population, a screening question, equal to the inclusion criterion, was used: ‘Do you work as a physician within oncology, urology, haematology, gynaecology, pulmonology or paediatrics, and treat patients with cancer?ʼ. Participation in the survey was voluntary, and informed consent was implied by the act of opening and responding to the questionnaire, as approved by the Regional Ethical Review Board.

### Analysis of the Quantitative Data

Quantitative data were analysed using IBM SPSS Version 24.0 (IBM Corp., Armonk, NY, USA) and StataCorp. 2019. Stata: Release 16. Statistical Software (StataCorp LLC, College Station, TX, USA). Descriptive statistics with frequencies and percentages were calculated for all questions/variables, including demographic data. To simplify analysis and enhance visualisation, the six-point Likert scales were grouped into three categories: Seldom (Likert scales 1-2), Sometimes (Likert scales 3-4) and Often (Likert scales 5-6).

### Analysis of the Open-Ended Responses

Open-ended responses generated by the participants relating to the aforementioned dimensions ‘Personal experiences’ and ‘The role of the organisation’ were analysed in a separate document. Thematic analysis with an inductive approach, inspired by Braun and Clarke,^26^ was used. Initially, two members of the research team (oncologist/palliative care specialist, KA, and paediatric oncologist, MS) independently read the answers (n = 417), which varied in length, to familiarise themselves with the content. In the next phase, codes capturing the physicians’ experiences were generated. The codes were then analysed and clustered into different sub-themes. These sub-themes were evaluated with the original text to assess their overall contribution to the data and determine whether they addressed the aim of the study. Finally, sub-themes that were related to each other were defined, resulting in three themes. Thereafter, the coding process and themes were discussed with a third member of the research team (registered nurse/professor, JM), after which consensus on the findings was sought. Verbatim quotations from the participating physicians were used to support the results. Finally, the findings were translated into English and checked by a qualified language reviewer.

The study was approved by the Regional Ethical Review Board in Linköping, Sweden (Registration number: 2016/47-31).

## Results

The results are presented with demographic data, and both the quantitative and the qualitative (open-ended responses) results present data from the dimensions ‘Personal experiences’ and ‘The role of the organisation’. Thematic analysis of open-ended responses, resulted in three themes based on the participating physicians’ view: ‘Navigating among barriers and facilitators, ‘Benefits of mutual collaboration in referrals’ and ‘Managing the organisational labyrinth’.

### Participants

In total, 239 presumptive physicians were identified and defined as the target population. The study questionnaire was answered by 130 (54.4%). More than half of the participating physicians were female (54.6%, n = 71). Gynaecological oncologists/gynaecologists (29.2%, n = 38) and oncologists (27.7%, n = 36) were the largest groups of medical specialists represented. The vast majority (78.1%, n = 100) consisted of specialists/senior consultants. The majority (59.3%, n = 70) had worked with cancer patients for more than 10 years. The study characteristics are shown in Table 1.

#### The physicians' personal experiences (Table 2) Personal Reflections and Patient-Physician Relations

When introducing PC to patients, most physicians in our study said that they felt confident in the dialogue (97.6%, n = 122) and acknowledged that the patient´s trust was preserved (99.2%, n = 125). Less than half of the physicians were afraid of making the patient upset (42.8%, n = 54) or seldom experienced feelings of unease before the task (45.2 %, n = 57). Additionally, the majority reported both medical (88.0%, n = 96) and emotional (74.6%, n = 79) relief when the care of the patient was enrolled in primary PC or specialised PC (92.7%, n = 102; 85.2%, n = 92 respectively).

#### Knowledge About PC Services

The physicians declared a high level of knowledge regarding the overall organisation of PC services in their county (95.3%, n = 120) and the criteria for determining the level of PC (82.2%, n = 101). Their source of information for organisational issues was colleagues (77.3%, n = 95), with digital sources playing a less significant role (37.6%, n = 47).

#### The role of the organisation (Table 3) Early PC and Its Influence on Oncology Care

The choice of oncological treatment remained unaffected when patients’ care was integrated into primary (89.4%, n = 93) or specialised PC (83.6%, n = 92). Nearly half (46.3%, n = 50) of the physicians reported that they did not receive information from the recipient that influenced their treatment decisions.

#### Organisational Challenges

Just over half of the responding participants (50.9%, n = 55) faced challenges in motivating the recipient about the necessity of early PC referral. Only a minority (12.6 %, n = 14) said that they perceived an unclear division of responsibilities. Almost one-third of the responding physicians did not actively obtain information (28.7%, n = 31) and rarely engaged in an active dialogue about the patient’s status with the recipient (30.3%, n = 33).

#### Opinions About PC Services

Opinions concerning the overall functionality of PC services varied. More than half of the physicians (52.4%, n = 66) were satisfied, while somewhat fewer (30.2%, n = 38) found the services acceptable, and only a small portion (12.7%, n = 16) were dissatisfied. Specialised PC received more positive evaluations (74.2%, n = 92) than primary PC (36.0%, n = 45). More than half of the participants (54.5 %, n = 66) expressed the opinion that equal access to PC varied and depended on where patients lived.

### Outcomes of Open-Ended Responses

The thematic analysis resulted in three themes and eight sub-themes which are illustrated with quotes.

#### Navigating Among Barriers and Facilitators

This theme illustrated challenges and enabling factors for the physicians in the referral process between oncological care and PC. The physicians reported different aspects that reflected the complexity of their task when they referred their patients to PC. They described factors that were connected to circumstances outside their control such as the individual patient’s specific situation and needs, and the organisation. It was also clear that the physicians’ personal situation affected their experiences, and both stressful elements and facilitators were identified. The theme consisted of three sub-themes: ‘Addressing communication and information challenges’, ‘Physician-related stressful elements’ and ‘Physician-related facilitators’.

*Addressing communication and information challenges* involved managing expectations and hope from the patients and their families. It addressed questions related to the patient’s wishes for oncological treatment and the challenge for the physician in getting the patient to accept the situation. The physicians expressed difficulties in providing balanced information to maintain hope, despite a poor prognosis, as well as managing potential disappointment when introducing and integrating PC.

“I can feel a challenge in getting across what I want to say clearly without sounding so depressing…” (Urologist, >10 years of experience)

The need for individualisation and for finding the right time to discuss PC, particularly with patients suffering from cancer with expected long-term survival, was considered crucial. Handling the reactions and expectations of next of kin was perceived as challenging, sometimes more than those of the patients themselves.

“To get next of kin to understand the situation can many times be more difficult than for the patient to accept the situation.” (Haematologist, ≤10 years of experience)

There were concerns regarding relationships and trust, illustrated by the fear of harming a patient-physician relationship and losing the patient’s trust, where the early introduction of PC was considered a potential risk. They said that the patient was seldom prepared for early referral. There was also a fear of giving the impression that the patient was being abandoned when referring them to PC. Introducing PC early in the course of the disease could be experienced as uncomfortable for the physician. Communication challenges related to the stigma of the words ‘palliative care ʼ, the need for a stepwise introduction of PC, and the importance of an individualised patient approach were emphasised.

“Sometimes you have to explain to the patient that it (referring to PC) doesn’t mean that you can’t give more treatment, that it doesn’t mean the end of life.” (Gynaecologist, ≤10 years of experience)

*Physician-related stressful elements* addressed aspects of physicians’ struggle with prognostic uncertainty for the individual patient, when to stop oncological treatment, and identifying when PC was indicated. In addition, collegial disagreement, and lack of consensus and communication between healthcare providers were identified as obstacles in the integration process. Different opinions about the best way to address patient’s needs and difficulties in gaining acceptance for early referral were highlighted. The physicians also expressed a lack of trust in PC providers concerning individual competence and interest, mainly regarding general PC providers. This, together with not knowing if the patient was going to be accepted for PC, led to uncertainty in the encounter with the patient.

“At times, it can be unsettling because one does not know how palliative care works in the context in which this patient resides. It may feel somewhat disingenuous to suggest that everything will be fine when, in reality, one lacks that knowledge” (Oncologist ≤10 years of experience)

The physicians’ report indicated that the respondents were well aware of the structural organisation of PC; however, this did not imply that they were knowledgeable about the functioning of individual units of PC. Concerns about their ability to provide enough support, personal distress about the patient’s situation, and the emotional burden of delivering bad news were also emphasised.

“… I am usually confident in the situation due to my long experience, but sometimes, it is very difficult to deliver these messages.” (Pulmonologist, >10 years of experience)

*Physician-related facilitators* illustrated factors that eased the physicians’ tasks in the referral process. To have had an established patient-physician relationship, with the opportunity to get to know the patient and build trust was considered to be valuable in the encounter with the patient and their next of kin when discussing PC.

“… If you have had a longer contact with the patient it is easier to know what quality of life means for this individual and consequently what is important during palliative care…” (Paediatric oncologist, ≤10 years of experience)

Long working experience was perceived as beneficial, leading to personal confidence in the situation when meeting the patients and their families. The ability to discuss patient issues within a team, leading to well-grounded decisions, was also regarded as valuable in contact with the patient.

“… I strive for clarity. I have often known the family for a long time, which generally alleviates issues of trust; furthermore, all decisions are made in consultation with paediatric oncologists at the regional level” (Paediatric oncologist, >10 years of experience)

### Benefits of Mutual Collaboration in Referrals

In this theme, supportive factors connected to the referral process between oncology and PC are described, giving a multi-faceted picture of the values that the physicians declared when referring patients to PC. This enrolment was perceived as beneficial not only for the patients and their next of kin, but also for the physicians on a personal level. The positive values for the healthcare system were illustrated and the possibility of achieving appropriate use of healthcare resources was highlighted. This theme was illustrated by three sub-themes: benefits for the physician, patient-related benefits, and identifying the optimal level of care.

*Benefits for the physician* covered the participating physicians’ experiences of both medical and emotional relief when the patients were enrolled in PC. Sharing the responsibility of patient care, and allowing PC providers to look after and take care of patients’ symptom control provided a sense of security and reduced the workload for the referring physician.

“[For me it] always feels good that there is someone helping the patient with symptom relief if I am not available.” (Pulmonologist, ≤10 years of experience)

Additionally, sharing the burden with PC colleagues helped to alleviate the stress for physicians regarding the risk of having patients with unmet needs, thereby preventing unnecessary suffering for their patients. Collegial confidence was demonstrated through the perceived benefits for patients who received support and frequent symptom monitoring from highly competent PC providers, highlighting the value of inter-professional collaboration in patient care. This collaboration was also considered beneficial on a personal level for the physician.

“[I have a] feeling of control if something suddenly worsens, feeling that we are joining together in a larger healthcare team around the patient… knowledge that difficult symptoms can be addressed together (one feels less lonely in the challenge).” (Oncologist, >10 years of experience)

*Patient-related benefits* encompassed the perceived benefits for patients and their families through the content of PC, where both emotional and physical symptoms were addressed by PC providers with competence, dedicating sufficient time for these concerns. In that way, they emphasised quality of life and at the same time helped patients and families to adapt to the situation.

“… I am convinced that patients needing PC receive better care in a palliative ward. Slower pace, hopefully, more time for dialogue and better pharmaceutical management, especially concerning palliative medicines.” (Urologist, >10 years of experience)

The benefits of effective symptom control—such as enabling patients to continue oncological therapy for a longer period—were also highlighted. The physicians pointed out that when care delivery was tailored to patients’ needs, involving PC and homecare services, it enabled patients to remain at home. This led to a decreased need for hospital contacts, emergency visits and hospitalisation. Established continuity of care could be achieved by reducing the number of healthcare providers involved and ensuring frequent contact with the patient at home. The possibility of home visits for regular monitoring of symptoms and adjustments in ongoing medication was considered beneficial.

“It is beneficial for the patient to have a readily accessible option for support without the need to go to the hospital.” (Gynaecologist, >10 years of experience)

*Identifying the optimal level of care* illustrated the advantages of PC involvement both in patient care and the utilisation of healthcare resources. By receiving information about the patient’s status and home situation from PC providers, the physician gained a clearer overall picture of the patient. This helped to identify patients with poor performance status that were not suitable for, or did not gain from, intensive oncological treatment.

“I get a better picture through the patient’s record of how the functionality is in the home between the visits in the hospital.” (Oncologist, ≤10 years of experience)

Early contact with PC could facilitate the process of ending oncological treatment when the time came, if the patient and next of kin already had an established relationship with PC providers.

…I am confident that the patient has become familiar with the team, which eases the cessation of oncological treatment when the time comes, and the patient and I know who is responsible for further care.” (Oncologist, >10 years of experience)

Optimising the utilisation of healthcare resources was also exemplified in how palliative homecare could address patients’ needs instead of requiring hospital care and resources.

“The patient receives assistance with medication adjustments, ie, painkillers and it reduces phone calls to our reception. The patient can receive quality care and there is a stable, supportive relationship.” (Urologist >10 years of experience)

### Managing the Organisational Labyrinth

This theme encompassed the role of the organisation in achieving successful integration between oncological care and PC. The physicians’ experiences of the role of the organisation highlighted the need for better ways of communication and coherence between healthcare providers involved in the process. The need for strengthening PC resources was also expressed. Limited resources among PC providers was highlighted as a problem for getting access to PC, and equal access for patients to PC was considered challenging. The theme was illustrated by two sub-themes: organisational opportunities and organisational challenges.

*Organisational opportunities* identified well-established communication pathways and transmission of information as key factors. Existing methods of exchanging information mostly involved reading the patients’ medical records except for paediatric oncologists, where oral communication was vital. A clear definition of personal responsibilities was considered important when the patient was referred to and enrolled in PC. Well-defined roles between physicians from different organisations facilitated cooperation in patient care.

“In early integration in PC, my judgement is that it is especially important to have frequent communication and clear demarcation concerning medical actions. When integration occurs in the end-of-life stage it is natural to leave a major part of the responsibility to PC.” (Urologist, >10 years of experience)

Paediatric oncologists actively stated that they wanted to maintain full patient responsibility, despite the outcome, ie, some of these physicians did not refer any patients to PC at all.

“The patient is never enrolled in any PC unit. It is my paediatric department or the home hospital (which are responsible). Some home hospitals are very experienced and handle most of the care themselves, but we continue to maintain a dialogue and update each other regarding shared care patients.” (Paediatric oncologist, ≤10 years of experience)

Individualisation, receptiveness to the patient’s wishes, and the need to keep the patient in focus during the referral process were emphasised.

“Early is good for diagnoses with a bad prognosis, but the patient’s needs and wishes also have to direct, for example, individuals living alone may require earlier support to ensure safety” (Oncologist, ≤10 years of experience)

*Organisational challenges* identified key factors affecting the achievement of equitable PC for all patients. These factors include geographical limitations, where patients residing in rural areas or counties may have restricted access to specialised PC and home care, as well as the local organisation of PC teams.

“For children, there continue to be significant disparities in what type of care they can receive at home depending on where they live.” (Paediatric oncologist, ≤10 years of experience)

Organisational shortcomings, encompassing insufficient resources and healthcare professionals among PC providers, were pointed out as problems associated with getting access to PC.

“Already when referring one gets the feeling that the referral is not going to be accepted due to lack of resources and prioritisation issues.” (Oncologist, ≤10 years of experience)

Resistance from PC providers was emphasised as a barrier to referral. This was expressed as reluctance to accept early referrals, referral criteria excluding patients with ongoing oncological treatment, and dissimilarities in opinions regarding responsibilities.

“…I would like earlier connection to specialised PC, but they are restrictive during active oncological treatment.” (Oncologist, ≤10 years of experience)

Some physicians also described a perceived deficiency in competence and interest among PC providers, ie, primary PC compared to specialised PC providers, as a cause of inequality.

“Those living near cities can access specialised PC and be helped by specialised physicians in the field. Others are supported by general practitioners with varying levels of expertise and engagement in PC.” (Oncologist, ≤10 years of experience)

## Discussion

In the present study, most of the physicians working with patients with cancer declared that they felt confident when discussing PC with their patients. They described professional, personal, patient-related and healthcare system-related benefits when patients were enrolled in PC. At the same time, the physicians tried to navigate among facilitators and barriers during the referral process, with the goal of balancing the patient’s needs with the relatives’ views and their own work situation. Mutual collaboration led to several advantages such as the sharing of responsibility, continuity and the utilisation of resources. More than half of the physicians stated that the patients did not have equal access to PC. To be able to achieve equitable PC, the importance of considering organisational aspects, including communication pathways and geographical restrictions, was highlighted.

The participating physicians reported multiple advantages when referring patients to PC. They reported feeling confident during the encounters with patients when discussing PC and said that the patients’ trust was preserved. These findings differ from the results of other studies that have described the introduction of PC as a demanding task.^9,16,22,27^ A potential explanation for our study findings could be that most of the participants were physicians with long experience of working with cancer patients, which may have led to the development of advanced communication skills. Physicians often experience distress when introducing palliative care due to insufficient training in communication.^16,28^ This illustrates the importance of education and training in communication when preparing physicians for the challenging task of meeting and handling patients who need PC.^16^ Even so, communication was viewed as a challenge for the participating physicians, particularly in delivering honest information about a disease with a poor prognosis, while simultaneously preserving patient hope. Expectations about the potential of oncological treatment and the possibility of a cure can be high among patients.^16,29,30^ The risk of depriving patients of hope, causing fear of abandonment, and determining the right time to discuss PC are known barriers to referral.^16,31^ It is important to be aware of the emotional effect these encounters with patients may have on the physician at a personal level in clinical work. Teamwork and an open work-related climate can serve as protective factors giving possibilities for debriefing, feedback and support, thereby reducing the risk of burnout for all team members.^19,20^ In the present study, the need for individualised care, attentiveness to patients’ wishes, and the importance of allowing patients time to come to terms with their situation were identified as key considerations. Determining the appropriate timing for referral based on the specific diagnosis was considered crucial, particularly given the challenge of prognostication, as some patients may live for many years with an incurable disease. These findings are in line with earlier studies and are known to be potential obstacles to the introduction and referral to PC.^30,32,33^ Engaging in early discussions about PC with patients who are not yet experiencing severe symptoms and are still responding to anti-cancer therapy is considered challenging and may contribute to delayed referrals. Educating healthcare providers, patients, and the wider public about the scope and objectives of PC, emphasising that it is not limited to end-of-life situations, may help to address some of the barriers to early referral.^16,30^

Some physicians perceived recommending early PC as burdensome and expressed concern that it might negatively impact the patient-physician relationship. Participants described having a long-term relationship with the patient as both a personal challenge and a facilitating factor in the transition from oncology care to PC. This finding is consistent with prior studies conducted among physicians working with patients with cancer.^9,16,34^ The physicians also expressed feelings of sadness regarding their patients’ situation and described the personal burden associated with delivering bad news. The challenge of balancing emotional connection with professional detachment in interactions with patients and their families has been highlighted in previous studies.^20,35^ Personal grief experienced among oncologists when patients deteriorate and die, along with the task of ‘breaking bad newsʼ^36^ are well-documented stressors for the physician. These challenges are associated with an increased risk of burnout,^16,37-39^ and the literature highlights the risk when being an oncologist.^40,41^ Sharing this emotional burden with PC colleagues may serve as a coping strategy in the demanding work of caring for severely ill patients and their relatives.^21,42^

The physicians emphasised that meeting and handling the expectations of relatives and their reactions, as well as using the words ‘palliative careʼ were perceived as challenges in the encounter with patients and their next of kin. These findings align with the results from previous research identifying similar factors as barriers for referral.^9,15,30,43^ Acknowledging these responsibilities as challenging, and being willing to seek support from colleagues, other healthcare professionals, or through organised supervision, may help alleviate stress and increase confidence in professional stances.^16^

Numerous study participants expressed views on the significance of organisational factors in the transfer process between oncology and PC. The vast majority of the physicians stated that both primary and specialised PC contributed to easing their own medical responsibility and emotional burden when caring for cancer patients. The involvement of PC providers, along with dedicated time and attention to improving quality of life, and addressing issues related to the concept of ‘total pain’,^44^ were perceived as beneficial for patients and their families. The physicians’ willingness to take sole responsibility for delivering PC was also expressed, particularly among paediatric oncologists, which in some cases led to delayed referrals or no referral at all. The desire of the physician to maintain full control over patient care, including PC, is a known barrier to referral.^35,45^ Contributing factors may include the physician’s perception that PC falls within their own responsibility, as well as structural aspects of how PC is organised.^9,22,46^ Moreover, access to PC is geographically unevenly distributed, with limited availability of paediatric PC expertise in certain regions.^47,48^ Ensuring equitable access to PC for both children and adults is a matter of national importance.^49^ In our study, many of the physicians reported having knowledge of both the organisation of PC in their county and the criteria for referral to specialised and general PC. Nevertheless, they expressed uncertainty regarding the local PC services. It is well established that limited knowledge about PC, along with variations in how local PC teams operate, can hinder the development of a systemic approach to early referral and enrolment in palliative home care.^22,32^ Previous studies have identified education in PC as a facilitator for early referral; however, concerns about organisational structure may still pose significant obstacles.^9,32^ The perceived variation in interest and competence in PC within primary care was emphasised as a challenge, contributing to physicians’ uncertainty when discussing PC with patients. This variation was also identified as a contributing factor to inequities in care. The physicians reported difficulties in gaining acceptance for early referrals to PC from the recipients. Diverging opinions among healthcare providers regarding patients’ needs, particularly the reluctance to offer PC to patients undergoing active oncological treatment, were highlighted as barriers in the referral process. Such disparities have also been shown in previous studies.^15,50^ Overall, these challenges reduce the likelihood of successful early enrolment in PC and hinder the goal of achieving integrated care. To support the referral process, there is a need not only for well-established guidelines but also for education, multidisciplinary teamwork and collaboration, consensus-building, and mutual trust in care providers.^16,22,50^ In addition, the participating physicians expressed concerns regarding the accessibility and provision of PC, particularly for patients residing outside the urban area. Inequities in access to PC have been demonstrated in studies based on Swedish registers and parental reports,^47,48^ as well as in studies from other parts of Europe and North America.^51,52^ The importance of equitable access to PC has been acknowledged by the Swedish government, the European Council and the International Association for Hospice and Palliative Care. This remains an area in need of further development and research.^47,49,53^ In the present study, the physicians expressed general satisfaction with the overall functioning of PC services. However, they placed greater value on specialised PC, highlighting the commitment and expertise associated with such units. Communication with PC colleagues was limited and the integration of PC had only a minor impact on the oncological treatment. Nevertheless, the information provided by PC teams was appreciated as it offered a more comprehensive understanding of the patient’s condition. This information occasionally led to modifications in oncological treatment, allowing for adjustments based on the patient’s current situation, and in some cases, supporting decisions to discontinue burdensome therapies. The limited influence of PC integration on end-of-life cancer treatment and the importance of effective communication between care providers have been documented in the literature.^22,54^ This underscores the importance of organisational efforts to develop effective communication channels and mechanisms for information exchange among healthcare providers, to ensure optimal care for each patient. Consistent with the findings of the present study, collegial trust, personal relationships, well-established communication pathways and inter-professional collaboration among healthcare providers are recognised as key facilitators in the referral and integration process.^22,50^

### Strengths and Limitations

The study was conducted using a regional approach, including participants from various medical specialities handling oncological treatment of patients with malignancies, which added nuances to the data. The use of an online questionnaire allowed rapid distribution and ease of response for the participant. Furthermore, allowing participants to provide open-ended responses to items with predetermined options enabled them to share nuanced, context-specific insights that may not have emerged through structured response formats alone.

There was a risk of response bias, as physicians with a particular interest in the topic and extensive experience may have been more likely to participate or may have provided socially desirable responses, which could have influenced the results. Additionally, the response rate was moderate, and there was an imbalance in the number of respondents across medical specialities. Some items in the questionnaire had varying degrees of missing data which may have influenced the results. These variations could potentially affect the completeness and representativeness of the findings, and should be considered when interpreting the results. The qualitative data were collected through written open-ended responses rather than interviews, potentially limiting the depth and richness of the insights obtained. Members of the research group interpreted the open-ended responses. Their pre-understanding may have influenced the analysis, potentially leading to overlooked nuances in the responses.

## Conclusion

The physicians in our study reported feeling confident when discussing PC with their patients. They acknowledged the value that PC has in patient care and the support it offers to relatives. However, they also described challenges in communication about PC and managing the emotional responses of patients and their next of kin. On a personal level, physicians found both emotional and professional relief when patients were enrolled in PC. The participants highlighted the risks posed by organisational shortcomings and disparities, which could negatively impact patients’ access to PC, ultimately contributing to unequal care. In our ongoing follow-up studies, PC providers will participate in order to more comprehensively reflect the significance of organisational factors in the referral process.

## Data Availability

In accordance with the ethical approval, the authors will not be sharing the data.*

## Appendix

### Abbreviations

PC: Palliative care
WHO: World Health Organisation.
